# Fish By-Product Valorization as Source of Bioactive Compounds for Food Enrichment: Characterization, Suitability and Shelf Life

**DOI:** 10.3390/foods11223656

**Published:** 2022-11-16

**Authors:** Adrián Honrado, Sara Rubio, José Antonio Beltrán, Juan Calanche

**Affiliations:** Instituto Agroalimentario de Aragón-IA2-(Universidad de Zaragoza-CITA), Miguel Servet 177, 50013 Zaragoza, Spain

**Keywords:** sea bass, shelf life, fatty acids, valorization, bioactive compounds

## Abstract

Fish processing generates many by-products, which are mainly destined for aquaculture feed. However, these by-products have interesting nutritional properties and could still be used for human consumption, thus promoting circular economy. Therefore, this study focused on evaluating the shelf life of mechanically deboned and dried meat (MDDM) of sea bass based on the lipid oxidation criterion (TBARS). The effect of a tocopherol-based antioxidant was also evaluated, and changes in the fatty acid profile were studied. For that, samples with and without antioxidant were stored at three temperatures (37, 55, and 65 °C) for 50 days. This allowed its modelling according to the Arrhenius model. The results showed a shelf life for MDDM of 220 days at 20 °C without the addition of antioxidant. When antioxidant was added, a high protective effect against oxidation and preservation of unsaturated fatty acids was perceived, avoiding nutritional losses and negative sensory effects, reducing EPA and DHA losses by 75% and 72%, respectively. In conclusion, the stability of MDDM from sea bass was demonstrated, making possible its incorporation into other food matrices.

## 1. Introduction

Fish is one of the most characteristic foods in several countries worldwide. According to FAOSTAT (The Food and Agriculture Organization Corporate Statistical Database) [1], some of the countries with the highest consumption can be found in Europe; Iceland (90.71 kg/per capita), Portugal (56.84), Spain (42.47), France (34.37), and Italy (29.80) are some of them. This is closely related to the Mediterranean Diet. Fish is an important part of this diet, which recommends consuming more fish (at least twice a week) than meat (monthly) [2]. Fish is characterized by its low cholesterol content and its high macro and micronutrient content: high biological value protein (16–21%) and fatty acids (0.2–25%) [3]. Some micronutrients such as vitamins A, D, and E also stand out [4,5].

Health benefits from fish consumption have been primarily related to polyunsaturated fatty acids (PUFAs), especially the omega 3 α-Linolenic acid (ALA), eicosapentaenoic acid (EPA), and docosahexaenoic acid (DHA) that can be found in large amounts in most fish [6]. EPA and DHA are important essential omega 3 fatty acids in the human diet as they are precursors of the 3-series eicosanoids, such as prostaglandins, thromboxanes, and leukotrienes, playing significant roles in immune function, inflammation, or thrombosis [7]. Proteins also play an important role in human health due to their beneficial biological activity on body functions including antimicrobial, antioxidant, and antihypertensive activities, among others [8]. However, fish is a product that causes rejection among young people, not only due to its taste, but also because of the difficulty involved in its cleaning and preparation compared to meat. About this, a study carried out in Spain on people aged between 25 and 35 found that 30% of young people consumed fish two or less times a week, 6% never consumed fish, and that after emancipation fish consumption declined significantly [9]. To facilitate its consumption, over the two past decades there has been a significant promotion of fish intake. This has led to the existence of new purchasing formats, as an alternative to traditional over-the-counter purchases [2], especially for some species such as sea bass or sea bream, whose world aquaculture production has increased [10]. 

The most immediate consequence has been the growth of fish processing, and the generation of significant amounts of fish by-products [11] such as whole fish or filleting trimmings, which can account for 55% of the total weight [12]. For this reason, there is a huge potential in converting fish processing waste into high-value nutritious products. The common destination of these products is aquaculture fish feed, being a direct option for the treatment of aquaculture by-products when a nearby facility is available. For example, in Chile, exports of salmon meal and oil for these purposes reached 105,000 t in 2016 [13]. However, this entails a devaluation of the product and even a series of costs derived from waste management. To add value to the product, other options are being considered [14]: mainly the production of food ingredients or the obtention of high-value biomolecules [12]. A clear example of this is the production of protein hydrolysates from these by-products to get bioactive peptides, as their biological activity and functional properties have been demonstrated; studies carried out on rats have shown beneficial effects on skin and bone health, on the lipid profile of the blood, and on weight control, which suggests that these benefits could be similar in humans [15,16,17]. Furthermore, the incorporation of lipids obtained from by-products into other foods, or their intake as a supplement (capsules, omega-3 concentrates, emulsions, and other formats), seems to be another option for revalorization.

In this regard, some research studies have evaluated the acceptability of bread produced by enriching wheat flour with tilapia protein flour in varying proportions, obtaining satisfactory results and consumer acceptability [18]. Mechanically deboned meat (MDM) from by-products has usually been used to produce fish burgers, nuggets, or breaded steaks [19]. Some studies suggest that using residues from filleting to produce MDDM (mechanically deboned and dried meat) resulted in a highly nutritious product with optimal physical, physicochemical, and microbiological characteristics [20]. Other studies have focused on the evaluation of the nutritional and technological potential of the addition of MDDM in traditional pasta [21,22] and in rice flour-based gluten-free pasta [23], with optimal results: high protein and unsaturated fatty acids content and low sensory modifications. In bakery, a kind of biscuit was produced with up to 40% edible fish meal, achieving high biological value protein contents of 15.52% and 22.5% fat, with a predominance of unsaturated fat [24]. Consequently, this translates into higher added value for the by-product and the promotion of circular economy.

Once its nutritional properties and the feasibility of incorporation in other products have been demonstrated, it is necessary to know its stability over time, both during storage and in the final product. This will depend on the composition, not being the same for a protein isolate as for a MDDM. The latter will contain unsaturated fat, which is susceptible to oxidation. As some authors claim, mechanical separation process could represent a new way to better exploit species of interest for European aquaculture and acquire new market niches, but oxidative processes during the treatment must be limited and kept under control [25]. Oxidation leads to a decrease in the nutritional value of fish and the generation of potentially harmful compounds for health [26]. For those reasons, the use of antioxidants may be of interest especially when they are incorporated before the oxidative process begins [27,28].

In industry, both synthetic and natural antioxidants are used. However, recent publications indicate that the use of these synthetic antioxidants in food is being re-evaluated in terms of their metabolic pathways and toxic, mutagenic, teratogenic, and carcinogenic effects at high concentrations [27,28,29]. For this reason, there is a growing interest in studying the use of natural antioxidants. These compounds are used as an α, β, γ, and δ isomers mixture, being α-tocopherol the most widely distributed and active. Different spice extracts have been tested in a variety of food products and lipid matrices to evaluate their antioxidant activity, obtaining positive results with the use of rosemary extract (*Rosmarinus officinalis* L.) due to its high content of phenolic compounds such as rosmarinic acid and carnosic acid, thus becoming a promising alternative to the use of synthetic antioxidants [27]. 

It is thus necessary to carry out a study to determine the shelf life of a MDDM according to the lipid oxidation criterion. However, although real time shelf-life testing brings optimal results, it has the disadvantage of being time-consuming, especially for long deterioration processes such as oxidation [30]. Therefore, accelerated shelf-life tests (ASLT) are desirable, as these techniques considerably shorten the process to obtain the necessary experimental data [31]. Some authors have found the degradation rate (k) at different storage temperatures [32,33]. For these, the Arrhenius model has been used to describe the temperature dependence through the activation energy (Ea) of the reaction [34]. Therefore, the aim of this research was to determine sea bass MDDM shelf life by studying the lipid oxidation kinetics according to the Arrhenius model, as well as to determine the variation of the fatty acid profile over time with and without the addition of a natural antioxidant, establishing its stability for use as an ingredient in the preparation of foodstuffs, especially those rich in bioactive compounds.

## 2. Materials and Methods

### 2.1. MDDM Manufacturing

The MDDM was produced from fresh sea bass (*Dicentrarchus labrax*) by-product from aquaculture maintained at 0 °C until use. The procedure described by Aínsa (2019) [35] was followed, with some modifications. First, the trimmings were placed in an 8% brine at 4 °C for one hour. This reduced the microbial load and favored subsequent dehydration. Then, the trimmings were placed on holed trays and placed in an oven (Mod. Junior 1100, Verinox, Altopiano della Vigolana, Italy) at 60 °C with slow air circulation for 24 h. Higher temperatures were not used to avoid modifying the fatty acid profile. After cooling until room temperature, the skin was removed and the muscle was minced (Mod. A320R1, Moulinex, Barcelona, Spain) and sieved (1.2 mm) to obtain a powder. Finally, the MDDM was vacuum packed (Mod. EV13, Tecnotrip, Barcelona, Spain) and stored at −20 °C until use.

### 2.2. Raw Material (By-Product) and MDDM Quality Parameters

#### 2.2.1. Moisture

The moisture content was determined by thermogravimetric analysis using an automatic thermobalance (Mod. DBS 60-3, KERN & Sohn GmbH, Balingen, Germany).

#### 2.2.2. Water Activity (aw)

This parameter was evaluated in an automatic water activity measuring device (Mod. CX-1, Decagon Devices, Inc., Pullman, WA, USA) using the protocol described in the user’s manual.

#### 2.2.3. Acidity Index

The determination of the acid index was carried out on each sample according to the Copant method [36]. A total of 2 g of sample was homogenized with petroleum ether (Carlo Erba, Milan, Italy). After evaporation, 10 mL of neutralized ethanol (VWR, Radnor, PA, USA) was added, and the sample was titrated with 0.1 N NaOH using phenolphthalein (Panreac, Barcelona, Spain) as an indicator. Results were expressed in g of oleic acid per 100 g.

#### 2.2.4. Oxidative Stability (TBARS)

The extent of lipid oxidation was evaluated by the 2-Thiobarbituric Acid Reactive Substances (TBARS) assay. This analysis was determined following the methodology of Pfalzgraf et al. [37]. For the MDDM, only 2 g of sample was weighted to correct water loses during dehydration. A curve pattern was performed using 1, 1, 3, 3-tetrametoxipropane (Sigma-Aldrich, St. Louis, MO, USA). The samples were extracted in an Ultraturrax device (T-25 basic, IKA-WERKE, Staufen, Germany) with 20 mL of 10% trichloroacetic acid. They were then centrifuged at 4000 rpm at 4 °C for 30 min. The reaction with thiobarbituric acid (Sigma-Aldrich) was carried out in a thermostatic bath (Unitronic 2000, J. P. Selecta, Barcelona, Spain) at 97 °C for 20 min. After cooling the samples, absorbance was measured at 532 nm in a spectrophotometer (5625 UV/VIS, UNICAM, Algés, Portugal). Results were expressed as mg of malondialdehyde (MDA) per kg of sample.

#### 2.2.5. Total Volatile Basic Nitrogen (TVB-N)

This measurement was carried out following the procedure established in Commission Regulation (EC) No 2074/2005 [38]. A distillation (VELP, Mod. UDK129, Usmate, Italy) unit was used for this purpose. Samples were extracted with percloric acid 6% (VWR). NH_3_ was collected in boric acid 3%. Titration was carried out on an automatic titrator (SI Analytics, Mod. TitroLine^®^ 5000, Mainz, Germany) with HCl 0.01 N (Carlo Erba).

#### 2.2.6. Fatty Acid Profile

This determination made it possible to observe probable changes in the fatty acid composition due to the MDDM obtention process [39]. Firstly, 10 g of raw material or 2 g of MDDM was homogenized with an Ultraturrax device (T-25 basic, IKA-WERKE) using different solvents: chloroform (Carlo Erba), methanol (Carlo Erba), potassium chloride (Panreac), and water. This mixture was centrifuged for 10 min at 4000 rpm (Mod. Universal 320 R, Hettich, Tuttlingen, Germany), and the fat was extracted from the top. After incorporating BHT (Sigma-Aldrich) as antioxidant, solvents were evaporated with nitrogen gas. Afterwards, methylation was performed using 0.03 g of this previous fat. This fat was mixed with an intern pattern, C23:0 (TCI), which does not caused interferences in the matrix. Then, 2 mL of hexane (Carlo Erba) and 1 mL of potassium hydroxide (Panreac) saturated solution in methanol was added. The upper phase was used. To analyze the fatty acid profile, a gas chromatograph (HP-6890 II, Hewlett-Packard, Palo Alto, CA, USA) was employed with a column SP-2380 (100 m × 0.25 mm × 0.20 µm). The temperature program was 140–165 °C at 3 °C/min during 10 min and 165–220 °C at 5 °C/min during 50 min. Fatty acid content was quantified as total area (%) of identified fatty acids.

### 2.3. Antioxidant Capacity Evaluation of of Different Antioxidant Compounds

Given the lipid nature of MDDM, with the presence of PUFA susceptible to oxidation, it is essential to use substances that prevent this process, as it is a factor that limits the shelf life of the product. However, as a preliminary step, it is necessary to know the in vitro antioxidant capacity of these products to choose the one with the highest antioxidant power. For this reason, the following analyses were carried out on the antioxidant products shown in Table 1. It should be noted that three of the products were not able for human food use, although they were analyzed to observe possible differences between them and those suitable for human use.

#### 2.3.1. Antioxidant Capacity DPPH

The analysis was carried out according to the method proposed by (Llorach et al. (2004) [40]. A standard line was prepared from the working solution of Trolox (Sigma-Aldrich) ranging from 0 to 60 µM, which is a water-soluble vitamin E analogue. A total of 0.1 g of each product was weighed, mixed with 9 mL of ethanol (VWR), and successive dilutions were made, adding to all 900 µL of DPPH (Sigma-Aldrich) solution 133 µM. The samples were incubated for 2 h in the dark and read in a spectrophotometer (UNICAM, 5625 UV/VIS) at 515 nm. The results were expressed as mg TE (Trolox equivalents)/g sample.

#### 2.3.2. Total Polyphenol Content

The analysis was carried out according to the method proposed by (Ordoñez et al. (2019) [41] with some modifications. A standard line of gallic acid (Sigma-Aldrich) was prepared. A total of 20 μL of extract was mixed with 1580 μL of water and 100 μL of Folin Ciocalteau 2 N solution (Merck, Rowey, NJ, USA) was added. After 1 min, 300 μL of 20% Na_2_CO_3_ (Panreac) was added and stored for 2 h in the dark. The absorbance was measured at 700 nm (5625 UV/VIS, UNICAM). Polyphenol content was expressed as g GAE (gallic acid equivalents)/100 g sample.

### 2.4. Accelerated Shelf-Life Study -ASLD-

The shelf life of MDDM based on fat stability and expressed as a direct function of lipid oxidation reactions was obtained by determining the TBARS index (as explained in Section 2.2.4) over the storage time of the product using the ASLD method [42].

The study during MDDM storage was carried out using 3 stoves (VWR, Mod. IncuLine 150R) at different temperatures, according to the “Schaal test” which is the usual methodology applied in these cases [43]. TBARS values determined every 2–3 days in samples stored at 37 °C, 55 °C, and 65 °C were used for kinetic degradation modelling.

Specifically, the Arrhenius model was applied, which allowed to obtain the relationship between oxidation rate constant, activation energy, and temperature [33,44]. The highest temperatures corresponding to 55 °C and 65 °C were chosen to establish the 10 °C difference needed to calculate the Q_10_, which indicates the number of times the rate of a deterioration reaction is modified when the temperature is varied by 10 °C [42], while the temperature of 37 °C corresponded to the extreme temperature that the product could reach due to an increase during storage at room temperature. Two sample treatments were selected for this experiment: T1 or control MDDM treatment and T2, whose MDDM contained 0.2% Nutrabiol^®^ (following manufacturer recommendations), as it was the one that reached a higher antioxidant capacity. Nutrabiol^®^ was added once the MDDM was ready. The different samples were prepared by introducing 15 g of each treatment in Petri dishes. The experiment extended until day 50.

The decay kinetics was modelled using the Arrhenius equation assuming that the reaction rate constant is a function of temperature. This was expressed by the formula:

(1) $$k=A\cdot e^{-\frac{Ea}{R\cdot T}}$$

By applying logarithms, it was possible to obtain the equation of a straight line whose slope is defined by the coefficient *Ea*/*R*:

(2) $$\ln k=\ln A-\frac{Ea}{R}*\frac{1}{T}$$

where:

*k*: rate constant (frequency of collisions resulting in a reaction)
*A*: Pre-exponential factor (an empirical relationship between temperature and rate coefficient)
*Ea*: activation energy for the reaction
*R*: universal gas constant (8.31 J⋅K^−1^⋅mol^−1^)
*T*: absolute temperature (Kelvin)

To determine the value of the unknowns, the TBARS values were plotted versus time for each of the temperatures. A linear adjustment was made, which allowed to obtain 3 equations, one for each temperature, and therefore 3 *k* values, corresponding to each temperature. The representation of the ln of the *k* values versus the 1/*T* ratio and its subsequent linear adjustment allowed to obtain the *Ea*.

On the other hand, taking as a reference a TBARS threshold value of 1.5 mg MDA/kg [45] and from the 3 equations determined for each temperature (2), it was possible to calculate the shelf life of the products at each of the temperatures. Representing the log_10_ of these times versus temperature, the following equations were obtained:

(3) $$Log^{shelf\;life}=a\cdot T-b$$

(4) $$Shelf\;life=10^{\left(b-a\cdot T\right)}$$

The quotient between shelf life at 55 °C and shelf life at 65 °C corresponded to Q_10_. Oxidative stability was also studied by determining the fatty acid profile at the beginning and end of the study and comparing it with the control treatment.

### 2.5. Effectiveness of the Antioxidant Compound

The fatty acid profile determination was carried out in raw material, but also on the MDDM at the beginning and at the end of the experiment developed in 2.4 for the 2 treatments and 3 temperatures established. This allowed to establish a relationship between the oxidation level and the loss of fatty acids, as well as the evaluation of the antioxidant role in the preservation of lipid composition.

### 2.6. Statistical Analysis

Results were analyzed with descriptive and inferential statistics. First, a univariate analysis was carried out to verify the normality of the data and detect outliers’ values. Once the previous step was executed, statistical analysis was performed by a linear mixed model that included the treatment, storage time, and its interaction as fixed effects. Approximate F-ratio tests for each fixed effect were conducted and critical value for a statistically important effect was taken at *p* < 0.05. Pairwise comparison between means was carried out using Fisher’s multiple comparisons test (LSD). All statistical modelling and presentations were constructed with XLSTAT (2016). It should be noted that all measurements were conducted in triplicate. 

## 3. Results and Discussion 

### 3.1. Antioxidant Capacity Evaluation of Different Antioxidant Compounds

**Figure 1:**
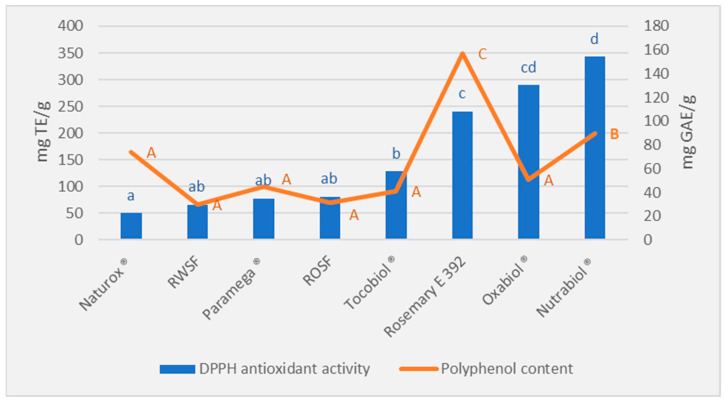
DPPH antioxidant activity and polyphenol content for different commercial antioxidant products. RWSF: rosemary water soluble fraction; ROSF: rosemary oil soluble fraction. Small letters indicate statistically significant differences (*p* < 0.05) among products for the DPPH antioxidant activity. Capital letters indicate statistically significant differences (*p* < 0.05) among products for the polyphenol content.

The results of the DPPH test of the different natural antioxidant compounds analyzed are shown in Figure 1. The compounds suitable for human consumption with the highest antioxidant power were rosemary extract (E 392) and Nutrabiol^®^, with significant higher performance (*p* < 0.05) in comparison to the rest of the antioxidants analyzed. Nutrabiol^®^ had the highest antioxidant capacity. It was followed by Oxabiol^®^, which has a similar composition, although is not intended for human consumption. An antioxidant activity of 217.75 mg TE/g was obtained in Wang et al. (2018) [46] for rosemary extract, which is a similar value to the one found in this study. Other studies agreed with these results as they show a high antioxidant power for rosemary extract, even more than antioxidants such as BHT [28].

The analysis of the polyphenol content of the different antioxidant compounds (Figure 1) revealed that the sample with the highest concentration of polyphenols is rosemary, being significantly higher than the rest (*p* < 0.05), and possibly responsible for its high antioxidant capacity. Similar values were obtained in Wang et al. (2018) [46] with 120 mg GAE/g. However, the rest of the antioxidant compounds have a low polyphenol composition, with no significant differences between them (*p* > 0.05), except for Nutrabiol^®^. Therefore, the high antioxidant capacity of the Nutrabiol^®^ compound is probably due to a combination of polyphenols and tocopherols, the latter substances characterised by long isoprenoid chains linked to methylated phenols [47] as the current trend is to mix different tocopherols with spice extracts rich in antioxidants with a polyphenolic structure [27].

Therefore, the antioxidant selected to carry out the study was Nutrabiol^®^, as the results showed the highest antioxidant capacity as well as being an additive applicable to food use.

### 3.2. Raw Material (By-Product) and MDDM Quality Parameters

Table 2 shows the results of the physicochemical parameters of both fresh by-product and MDDM.

Moisture and aw values in the by-product were found to be normal for fresh sea bass [48]. Due to the dehydration process, statistically significant differences (*p* < 0.05) were found between the by-product and the MDDM.

The acidity index showed a value of 0.092 g oleic acid/100 g, similar to that obtained for trout [49]. This index increased to 0.63 g oleic acid/100 g in the MDDM, considered as the limit of acceptability. This free fatty acid content may be due to the fact that the application of heat dehydration promotes their release from the triacylglyceride [50]. These results indicate the beginning of hydrolytic rancidity and therefore a certain availability of free fatty acids to be oxidized. 

However, the TBARS index value was 0.11 mg MDA/kg in fresh by-product, demonstrating the absence of lipid oxidation, which was very similar to the value published in other studies with fresh sea bass fillets [51,52]. This, together with the TVB-N value, indicates proper preservation and handling, which translates into optimal by-product quality. In the MDDM, the TBARS value was 0.19 mg MDA/kg, with no significant difference with the by-product (*p* > 0.05). Therefore, although heat produced the release of free fatty acids, the MDDM production process did not increase lipid oxidation.

In relation to the TVB-N, a value of 23.51 mg/100 g was obtained in the by-product, not exceeding the limit of 35 mg/100 g specified in Regulation (EC) No. 2074/2005 [38]. This value is also lower than that proposed by some authors who establish a specific quality limit for this species of 25 mg/100 g [53]. These low TVB-N values are a consequence of low post-mortem autolysis and bacterial degradation [5]. In the case of MDDM, the TVB-N value was 56.7 mg/100 g, which did not exceed the limit of 60 mg nitrogen/100 g laid down in Regulation (EC) No 1022/2008 [54] for whole fishery products used directly in the preparation of fish oil intended for human consumption. 

Regarding fatty acid profile, the results obtained on raw material-fresh by-product-(Table 3) showed ΣSFA, ΣMUFA, and ΣPUFA values of 21.8%, 49,06%, and 35.37%, respectively, like other studies on farmed sea bass such as the one carried out by Munekata et al. (2020) [55] where the addition of SFA, MUFA, and PUFA was 25%, 41%, and 32%, respectively. The research carried out, in the same species, by Baki, Gonener, and Kaya (2015) revealed a lower MUFA content of 27,6% in its composition due to fattening [56]. This variation could be due to a higher content of plant-based ingredients, such as soy, in the diet of farmed sea bass [57].

The results showed that palmitic acid (15.37%) and oleic acid (34.60%) were the most abundant SFA and MUFA, respectively. In relation to PUFA, the content of the ω-3 series was quite like that of the ω-6, with ALA (C18:3 ω-3), EPA (C20:5 ω-3), and DHA (C22:6 ω-3) predominating among the ω-3, which constitute 43.79% of this group and were the ones of greatest interest due to their several health benefits. However, the predominant PUFA was linoleic acid (C18:2 ω-6), which made up 42.49% of this group. This high value could be due to a high content of vegetable flours in the diet, mainly soy [58]. The predominance of the same fatty acids within each group is observed. However, a higher content of SFA to the detriment of MUFA is perceived which could be again due to the diet. The PUFA content was found to be similar. The Ω-3/Ω-6 ratio was close to 1, indicating a high nutritional quality product. This was higher than the relation found in [59] for sea bass fillet by-product, that was 0.67.

### 3.3. Accelerated Shelf-Life Study -ASLD-

**Figure 2:**
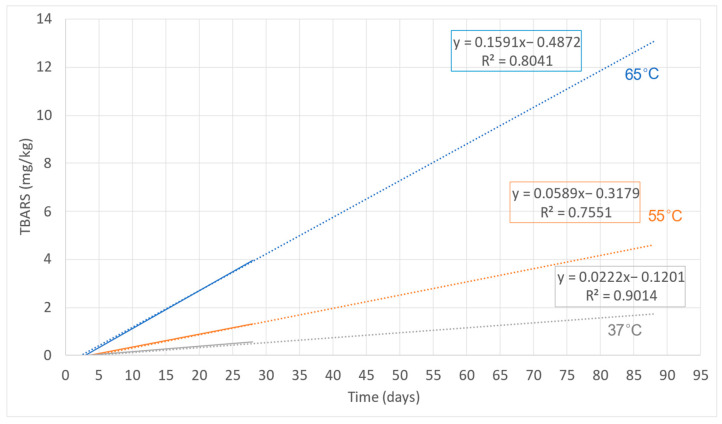
Linear adjustment and projection of TBARS values obtained in the MDDM at different days and temperatures (37, 55, and 65 °C).

Figure 2 shows the linear regressions obtained from the TBARS measurements made on treatment T1 over time at the three set temperatures (37, 55, and 65 °C). This allowed the three equations to be obtained (Equations (5)–(7)), one for each treatment temperature.

(5) $$TBARS^{37{}^{\circ}C}=0.0222\cdot t-0.1201$$

(6) $$TBARS^{55{}^{\circ}C}=0.0589\cdot t-0.3179$$

(7) $$TBARS^{65{}^{\circ}C}=0.1591\cdot t-0.4872$$

**Figure 3:**
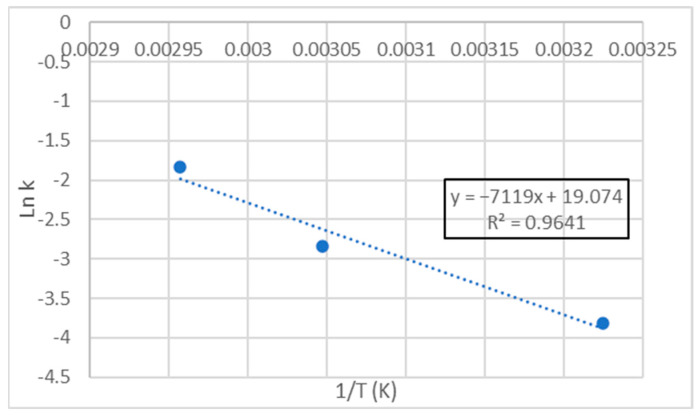
Linear adjustment of Ln k versus 1/T for TBARS in treatment T1.

The slopes of these equations, that corresponded to the reaction rate constant (*k* in Equation (2)), were considered to apply the Arrhenius Model, representing the ln *k* as a function of 1/*T*. A linear adjustment was made again. The resulting graph (Figure 3) and equation (Equation (8)) are shown below.

(8) $$\ln\;k=19.064-7112.4\cdot \frac{1}{T}$$

Once the value of the Ea/R ratio was obtained, considering the value of R, Ea was calculated, which turned out to be 59158.9 J/mol. This was the minimum required by the system before the oxidative process could start. This is a specific value for each product and remains constant as the temperature increases. Some authors indicate that the activation energy for lipid oxidation reactions is in the range of 41,842 J/mol to 104,605 J/mol, so the result is between the aforementioned values [60]. The Q_10_ was 1.8, which means that the oxidative process of MDDM is accelerated 1.8 times for every temperature increase of 10 °C. This value was similar to the ones obtained in other research studies [33,42].

**Figure 4:**
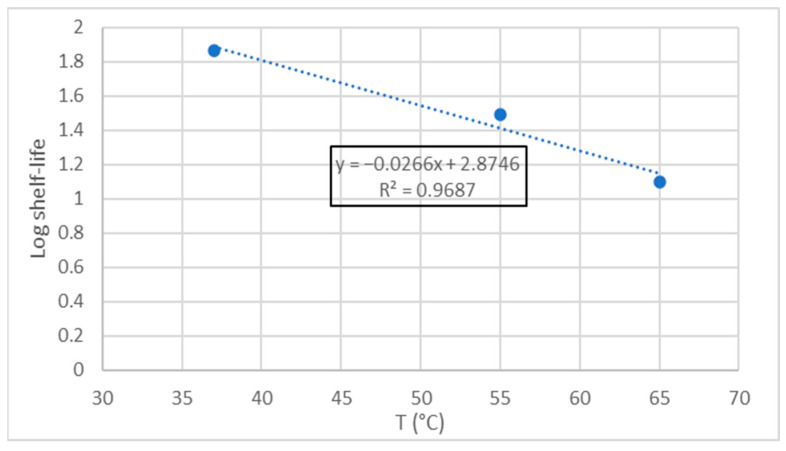
Representation of the shelf-life log as a function of temperature.

To determine the shelf-life value in relation to oxidative stability, a value of 1.5 mg MDA/kg was established as the limit of sensory acceptability, as some authors indicate that TBARS values above 1–2 mg/kg in fish are indicative of organoleptic and sensorially appreciable rancidity [45]. With this value, the shelf life of the MDDM at the three different temperatures were estimated for each temperature using Equations (5)–(7). Thus, the logarithm of the shelf life obtained was plotted against temperature (Figure 4), obtaining by linear regression Equations (9) and (10) which makes it possible to determine the shelf life of sea bass MDDM at different storage temperatures.


(9) Log shelf life=2.8746−0.0266·T

(10) $$Shelf\;life=10^{\left(2.8746-0.0266\cdot T\right)}$$

From Equation (10) it was possible to determine the best before date based on the lipid oxidation of the MDDM at the three temperatures of the experiment. This lifetime was found to be 78, 26, and 14 days for 37, 55, and 65 °C, respectively. Since this shelf life is calculated exclusively based on the lipid oxidation criterion, it is necessary to take into account that no other process will affect the shelf life of the MDDM and that the oxidative process will only be affected by temperature and not by other factors such as light presence or oxygen. This would require vacuum packaging that preserves the product from these ambient agents [59].

Therefore, assuming a storage temperature of 20 °C, the shelf life would be extended to 220 days. If refrigeration is applied and the product is kept at 4 °C, the shelf life would be 586 days. However, when manufacturing a food product with this type of MDDM, it must be considered that the shelf life of the new product will be limited by the remaining shelf life of the MDDM from the moment it has been manufactured and it would be advisable not to exceed the estimated shelf life of the ingredient to avoid organoleptic quality losses in the final product.

### 3.4. Effectiveness of the Antioxidant Compound

**Figure 5:**
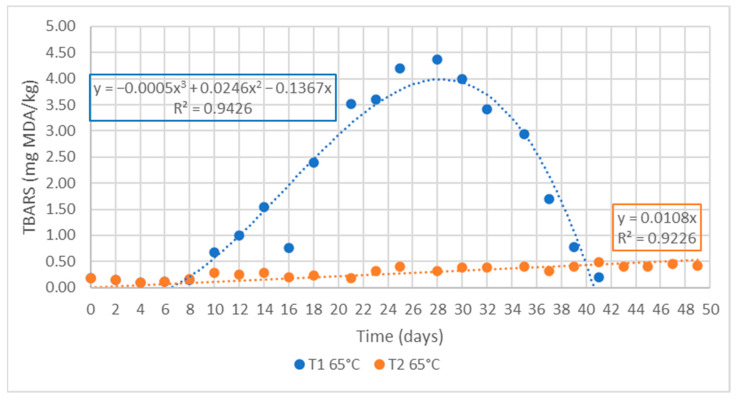
TBARS values obtained at T1 (control MDDM) and T2 (MDDM + 0.2% Nutrabiol^®^) during the accelerated shelf-life assay as a function of time at 65 °C.

Figure 5 shows the evolution of TBARS at 65 °C for treatments T1 and T2 (0.2% Nutrabiol^®^)

The effectiveness of the natural antioxidant compound added to delay the lipid oxidation process can be appreciated. In T1, the maximum value of the oxidative process was reached on day 28, with a TBARS of 4.2 mg/100 g. From now on, the concentration of MDA began to fall because of two effects: the depletion of free radicals producing this substance and the transformation of MDA into other simpler compounds. Sample T2, on the other hand, still presented a value close to 0.4 mg/100 g. Moreover, it remained stable until the end of the experiment at the maximum preset time, corresponding to a shelf life approximately three times longer than that determined in the T1 at 65 °C in the study of the kinetics of the oxidative process (Section 3.3). The high efficacy demonstrated by this natural antioxidant coincides with the results obtained in sardines [61], where the synergistic effect of α-tocopherol and rosemary extract showed a high efficacy, being greater than that of some synthetic antioxidants commonly used in this type of food, such as BHA. As can be seen in Figure 5, the maximum TBARS value during the study for T1 was reached at day 28, while for T2 it was at day 50.

### 3.5. Comparative Study in Fatty Acids Profile

The final sampling point (day 50) was selected for a comparative study of fatty acids profile among treatments T0, T1, and T2 (Table 3). The temperature of 65 °C was selected because it allowed to appreciate better the differences of the applied conditions on the state of the fatty acids. As indicated by some authors, the optimal working temperature of lipases could be found in the range between 35°and 50 °C, although there are thermostable lipases that exhibit optimal temperature values above 50 °C [62]. This could lead to an increase of hydrolysis reactions speed, releasing a greater amount of free fatty acids, susceptible of oxidation [62].

The analysis of the fatty acid profile of the developed product (T0) shown in Table 3 revealed a composition quite like the raw material (fresh by-product). No differences (*p <* 0.05) were found either in the most important groups of indicators (∑ SFA, ∑ MUFA, ∑ PUFA) or in the composition of fatty acids of interest such as ALA (C18:3 n-3), EPA (C20:5 ω-3), DHA (C22:6 ω-3), or α-linolenic acid (C18:3 ω-3). It could be concluded that the technological process developed to obtain MDDM did not affect the fatty acid profile.

The heat effect of incubation time at 65 °C could be observed when comparing T0 and T1. On T1, a significant increase (*p* < 0.05) of 42.6% and 9.37% in the percentage of SFA and MUFA could be observed. However, this increase would not be due to a rise in SFA or MUFA concentration, as it could be related to a decrease in PUFA. 

About them, since PUFA are the most sensitive to the oxidative process, their percentage decreased significantly (*p* < 0.05) at the end of storage at 65 °C, with a loss of 42.46%. Certainly, ω-3 and ω-6 acids followed the same trend, with a decrease of 68.42% and 48%, respectively. The decrease in EPA (C20:5 ω-3) and DHA (C22:6 ω-3) fatty acids was 89.14% and 92.16%, although a decrease of 45.2% of linoleic acid (C18:2 ω-6) and 34.95% of α-linolenic acid (C18:3 ω-3) took also place, being both significant (*p* < 0.05).

On the other hand, when comparing T0 to T2, the effect of the addition of the antioxidant on the fatty acid profile could be observed. At T2, in contrast to T1, the SFA content remained stable. However, MUFA content showed a decrease (*p* < 0.05) of 10.39%. This could be mainly related to gadoleic acid (C20:1). As it is constituted by a long monounsaturated chain and has a cis structure, its behavior is like PUFA and therefore more susceptible to degradation [63].

Related to the above, in the case of PUFA, there was only a decrease of 5.1%. ω-3 acids only decreased 5.21% and for ω-6, the application of antioxidant makes its composition remain stable. No changes were observed in either α-linolenic acid (C18:3 ω-3) or linoleic acid C18:2 ω-6 (*p* > 0.05) but there was a decrease in EPA (C20:5 ω-3) and DHA (C22:6 ω-3) of 14.21% and 20.96%.

The differences in the behavior of PUFA between T1 and T2 could be due to the addition of the antioxidant. PUFA are more predisposed to oxidation because of their large number of double bonds and their position within the chain. These characteristics mean that they have low activation energy for hydrogen loss and the formation of free radicals. The antioxidant added in T2 was able to neutralize these radicals [48]. In contrast, monounsaturated fatty acids and saturated fatty acids do not have the same characteristics and are therefore less affected [62]. Since DHA (C22:6 ω-3) shows the highest decrease rates, it could be claimed that it is the most susceptible fatty acid in the MDDM obtained from farmed sea bass. These results agree with those reported by Stéphan et al. (1995) [64], which demonstrated that the DHA was more labile and confirmed how the application of antioxidants composed by tocopherol prevented the reduction of the fatty acids of greatest interest by approximately 75 and 72%, respectively. In this sense, similar findings to those obtained for T2 in this experience were reported by Pirini et al. (2000) [65] in the flesh of farmed sea bass where fatty acid composition remained stable over time when fish were fed with alfa-tocopherol acetate supplemented diets. Some studies carried out in salmon [66] and turbot [67] also demonstrated the effect of alpha-tocopherol in preventing lipid oxidation.

Definitely, the results obtained show clear evidence of the effectiveness of the addition of this antioxidant composed mainly of tocopherols in delaying the oxidative process and protecting polyunsaturated fatty acids, since all the results obtained showed significant differences in the content of these (*p* < 0.05) concerning the values of the control sample (T1). 

## 4. Conclusions

This study has demonstrated that although MDDM is derived from sea bass trimmings resulting from processing, if proper cold storage and handling is applied, an edible product with high quality, nutritional value, and stability could be achieved. In this sense, the shelf life of the MDDM studied could be 226 days at 20 °C and absence of light and oxygen. The activation energy value and Q_10_ required to initiate the oxidation process were like the ones found in the bibliography. Furthermore, by using antioxidants, such as Nutrabiol^®^, it was possible to avoid the initiation of oxidation until the end of the experiment, as well as significantly decreasing (*p* < 0.05) the loss of the fatty acids of greatest interest: EPA and DHA. The addition of this antioxidant reduced the losses of these fatty acids by 75 and 72%, respectively, compared to the control sample.

## Figures and Tables

**Table 1:** Composition of the different products whose antioxidant capacity was evaluated.

Antioxidant	Composition
Rosemary E 392 (Marbys, Barcelona, Spain)	Rosmarinic Acid (8% min.), Carnosic acid (5% min.)
Rosemary water soluble fraction (RWSF) (Evesa, Cádiz, Spain)	Carnosic acid (5.5% min.)
Rosemary oil soluble fraction (ROSF) (Evesa, Cádiz, Spain)	Carnosic acid (5.7% min)
Tocobiol^®^ (BTSA, Madrid, Spain)	Tocopherols E 306 (18.5%), Sterols (<10%), Squalene (<5%), Monoglycerides (19%)
Paramega^®^ * (Kemin, Des Moines, IA, USA)	Tocopherols E 306, Botanical origin oils
Naturox^®^ * (Kemin, Des Moines, IA, USA)	Vegetable oil Tocopherols E 306, Lecithin, Rosemary extract E 392
Oxabiol^®^ * (BTSA, Madrid, Spain)	Tocopherols E 306 (50%), α-tocopherol (5% min.), β + γ-tocopherol (55% min.), δ-tocopherol 18% min.)
Nutrabiol^®^ (BTSA, Madrid, Spain)	Tocopherols E 306 (50%) α-tocopherol (13.8%), β + γ-tocopherol (58.4% min.), δ-tocopherol (27.8% min.)

* Usage in human-destinated foods is forbidden.

**Table 2:** Physicochemical properties of fresh by-product and MDDM.

Parameter	Fresh by Product	Mddm
Moisture (%) *	82.20 ^b^	23.08 ^a^
a_w_ *	0.9985 ^b^	0.9135 ^a^
Acidity index (g oleic acid/100 g) *	0.092 ^a^	0.63 ^b^
TBARS mg MDA/kg	0.11 ^a^	0.19 ^a^
TVB-N (mg/100 g) *	23.51 ^a^	56.71 ^b^

Different letters in the same row indicate statistically significant differences between samples (* *p* < 0.05).

**Table 3:** Fatty acid composition of fresh seabass (FSB), MDDM at day 0 (T0), MDDM at the end of the experiment at 65 °C (T1), and MDDM with 0.2% Nutrabiol ^®^ at the end of the experiment at 65 °C (T2). The end of the experiment was at day 50. Different letters in the same row indicate statistically significant differences between samples (* *p* < 0.05).

Fatty Acid	FSB	T0	T1	T2
C14	2.35 ^a^	2.39 ^a^	3.59 ^b^	2.56 ^a^
C15 *	0.25 ^a^	0.24 ^a^	0.35 ^b^	0.25 ^a^
C16 *	15.37 ^a^	15.27 ^a^	21.94 ^b^	15.76 ^a^
C17 *	0.28 ^b^	0.22 ^a^	0.37 ^c^	0.27 ^ab^
C18	3.25 ^a^	3.45 ^b^	4.77 ^c^	3.46 ^b^
C19	0.07 ^a^	0.07 ^a^	0.06 ^a^	0.04 ^a^
C20 *	0.20 ^a^	0.23 ^b^	0.31 ^c^	0.23 ^b^
C21*	0.00 ^a^	0.00 ^a^	0.00 ^a^	0.00 ^a^
C22 *	0.03 ^a^	0.08 ^a^	0.06 ^a^	0.04 ^a^
*∑ SFA*	21.79 ^a^	21.95 ^a^	31.45 ^b^	22.62 ^a^
C14:1	0.02 ^a^	0.03 ^a^	0.00 ^a^	0.00 ^a^
C16:1 *	3.85 ^a^	3.86 ^a^	4.96 ^c^	4.10 ^b^
C17:1	0.34 ^ab^	0.27 ^ab^	0.19 ^a^	0.49 ^b^
tC18:1 n-9 *	0.24 ^a^	0.31 ^a^	0.43 ^b^	0.32 ^a^
C18:1 n-11 *	3.06 ^b^	2.95 ^a^	3.75 ^c^	3.05 ^b^
C18:1 n-9 *	34.60 ^a^	34.67 ^a^	43.23 ^c^	35.26 ^b^
tC18:1 n-7	0.11 ^ab^	0.10 ^a^	0.13 ^c^	0.11 ^b^
C20:1	6.45 ^c^	6.43 ^c^	0.33 ^b^	0.25 ^a^
C22:1 n-9 *	0.33 ^b^	0.29 ^a^	0.38 ^c^	0.27 ^a^
C24:1	0.07 ^a^	0.07 ^a^	0.17 ^a^	0.03 ^a^
*∑ MUFA*	49.06 ^b^	48.97 ^b^	53.56 ^c^	43.88 ^a^
C18:3 n-3 ALA *	6.45 ^b^	6.43 ^b^	4.17 ^a^	6.32 ^b^
C18:3 n-6 *	0.23 ^a^	0.23 ^a^	0.03 ^a^	0.23 ^a^
tC18:2 n-6 *	0.30 ^b^	0.30 ^b^	0.16 ^a^	0.31 ^b^
C18:2 n-6 *	15.03 ^b^	15.95 ^c^	8.77 ^a^	16.04 ^c^
C20:2 n-6 *	1.18 ^c^	1.08 ^b^	0.61 ^a^	1.07 ^c^
C20:3 n-3	0.29 ^b^	0.30 ^b^	0.00 ^a^	0.44 ^b^
C20:3 n-6 *	0.23 ^b^	0.22 ^b^	0.03 ^a^	0.21 ^b^
C22:2 n-6 *	0.57 ^b^	0.52 ^b^	0.13 ^a^	0.30 ^ab^
C20:4 n-6 *	0.44 ^bc^	0.46 ^c^	0.03 ^a^	0.39 ^b^
C22:6 n-3 DHA *	5.47 ^d^	4.93 ^c^	0.38 ^a^	3.94 ^b^
C20:5 n-3 EPA *	3.57 ^c^	3.59 ^c^	0.39 ^a^	3.10 ^b^
C22:5 n-3 *	1.60 ^d^	1.26 ^c^	0.28 ^a^	1.10 ^b^
Ω3	17.38 ^d^	16.51 ^cd^	5.21 ^a^	14.91 ^b^
Ω6	17.98 ^b^	18.77 ^c^	9.76 ^a^	18.57 ^bc^
*∑ PUFA*	35.37 ^c^	35.28 ^c^	14.98 ^a^	33.48 ^b^
*∑ Total UFA*	84.42 ^c^	84.25 ^c^	68.53 ^a^	77.35 ^b^
*∑ Total UFA + SFA*	106.21	106.20	99.98	99.97

## Data Availability

Data is contained within the article.
